# The relaxin family peptide receptor 1 (RXFP1): An emerging player in human health and disease

**DOI:** 10.1002/mgg3.1194

**Published:** 2020-02-26

**Authors:** Ting‐Yun Chen, Xiaoyun Li, Ching‐Hsia Hung, Harinath Bahudhanapati, Jiangning Tan, Daniel J. Kass, Yingze Zhang

**Affiliations:** ^1^ Division of Pulmonary, Allergy and Critical Care Medicine and the Dorothy P. and Richard P. Simmons Center for Interstitial Lung Disease University of Pittsburgh Pittsburgh PA USA; ^2^ Institute of Allied Health Sciences National Cheng Kung University Tainan Taiwan; ^3^ Department of Human Genetics University of Pittsburgh Pittsburgh PA USA

**Keywords:** alternative splicing, fibrosis, relaxin, RXFP1

## Abstract

**Background:**

Relaxin/relaxin family peptide receptor 1 (RXFP1) signaling is important for both normal physiology and disease. Strong preclinical evidence supports relaxin as a potent antifibrotic molecule. However, relaxin‐based therapy failed in clinical trial in patients with systemic sclerosis. We and others have discovered that aberrant expression of RXFP1 may contribute to the abnormal relaxin/RXFP1 signaling in different diseases. Reduced RXFP1 expression and alternative splicing transcripts with potential functional consequences have been observed in fibrotic tissues. A relative decrease in RXFP1 expression in fibrotic tissues—specifically lung and skin—may explain a potential insensitivity to relaxin. In addition, receptor dimerization also plays important roles in relaxin/RXFP1 signaling.

**Methods:**

This review describes the tissue specific expression, characteristics of the splicing variants, and homo/heterodimerization of RXFP1 in both normal physiological function and human diseases. We discuss the potential implications of these molecular features for developing therapeutics to restore relaxin/RXFP1 signaling and to harness relaxin's potential antifibrotic effects.

**Results:**

Relaxin/RXFP1 signaling is important in both normal physiology and in human diseases. Reduced expression of RXFP1 in fibrotic lung and skin tissues surrenders both relaxin/RXFP1 signaling and their responsiveness to exogenous relaxin treatments. Alternative splicing and receptor dimerization are also important in regulating relaxin/RXFP1 signaling.

**Conclusions:**

Understanding the molecular mechanisms that drive aberrant expression of RXFP1 in disease and the functional roles of alternative splicing and receptor dimerization will provide insight into therapeutic targets that may restore the relaxin responsiveness of fibrotic tissues.

## Graphical abstract

The failure of antifibrotic effects in systemic sclerosis patients by relaxin may be the result of reduced expression of its receptor relaxin family peptide receptor 1 (RXFP1) in the fibrotic tissues. Alternative splicing and RXFP1 dimerization with other receptors also play roles in regulating relaxin/RXFP1 signaling. Molecular mechanisms underline the reduced RXFP1 expression and alternative splicing are potential therapeutic targets for restoring relaxin sensitivity in fibrotic diseases.

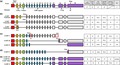

## 1 INTRODUCTION

The Relaxin/relaxin family peptide receptor 1 (RXFP1) axis is an “old” pathway (Bennett, 2009; Chihal & Espey, 1973) and the idea that relaxin's actions could be harnessed as an antifibrotic emerged from the seminal work in 1926 identifying relaxin as a hormone that could relax pelvic ligaments (Fevold, Hisaw, & Meyer, 1930; Hisaw, 1926). More recent studies suggest that aberrant expression of RXFP1—with its potentially negative consequences on relaxin signaling—is an important contributor to several diseases (Bahudhanapati et al., 2019; Corallo et al., 2019; Fallowfield et al., 2014; Feng & Agoulnik, 2011; Feng et al., 2010; Giordano et al., 2012; Nagorniewicz et al., 2019; Tan et al., 2016; Thanasupawat et al., 2019). RXFP1 expression can be modulated, and alternative mRNA splicing transcripts may have potential functional consequences in disease tissues (Bahudhanapati et al., 2019; Chow et al., 2014; Chow et al., 2019; Fagerberg et al., 2014; Hsu et al., 2000; Kern & Bryant‐Greenwood, 2009; Kern, Hubbard, Amano, & Bryant‐Greenwood, 2008; Muda et al., 2005; Sasser, 2014; Scott et al., 2006; Scott, Tregear, & Bathgate, 2005; Tan et al., 2016). Therefore, this review will focus on the aberrant expression, functional alterations associated with mRNA splicing variants, and posttranslational heterodimerization of RXFP1 in human physiology and disease. We will discuss the potential implications of the abnormal RXFP1 changes in developing therapeutics to restore relaxin/RXFP1 signaling.

The relaxin family peptide receptor 1 (RXFP1) mediates relaxin‐2 (relaxin) signaling (Hsu et al., 2002). A total of four relaxin receptors, RXFP1 to RXFP4, have been identified. All four are members of the class A seven‐transmembrane G‐protein‐coupled receptor (7TM GPCRs) superfamily based on sequence homology and functional similarity (Banerjee & Mahale, 2015; Kleinlogel, 2016; Yegorov, Bogerd, & Good, 2014). RXFP3 and RXFP4 are classical peptide receptors with a short N‐terminus extracellular domain, while RXFP1 and RXFP2 contain a leucine‐rich repeat (LRR) domain and a low‐density lipoprotein class A (LDLa) module in their extracellular region and belong to the LRR‐containing G protein‐coupled receptor (LGR) subfamily (Bathgate et al., 2013; Yegorov et al., 2014). The extracellular domain of RXFP2 mediates the effects of insulin‐like peptide 3 (INSL3) (Halls et al., 2005; Wilkinson, Speed, Tregear, & Bathgate, 2005). Although relaxin and INSL3 both activate RXFP1 and RXFP2 in vitro, there is no evidence that RXFP2 is activated by relaxin in vivo (Hsu et al., 2002; Kumagai et al., 2002; Scott, Fu, et al., 2005). Moreover, the linker in RXFP2 lacks the proposed binding region for relaxin and thus has a lower affinity for relaxin than RXFP1 (Hoare et al., 2019). The relaxin/RXFP1 system has a much wider range of tissue distribution and function than INSL3/RXFP2 (Halls, Bathgate, & Summers, 2006; Halls, Bathgate, Sutton, Dschietzig, & Summers, 2015).

## 2 RELAXIN/RXFP1 SIGNALING

Relaxin is a heterodimeric peptide hormone with a two‐chain structure (Wilkinson et al., 2005). It was first identified by Frederick Hisaw in a guinea pig model of pregnancy and parturition (Fevold, Hisaw, & Meyer, 1930; Hisaw, 1926). Relaxin was observed to loosen pelvic ligaments to facilitate parturition by reducing the density of collagen bundles and *relaxing* the collagen fibers (Chihal & Espey, 1973; Hisaw, 1926; Wilkinson et al., 2005). Additional roles of relaxin/RXFP1 signaling axis were identified in many physiological processes including development of mammary nipples and vaginal epithelium in mice (Kaftanovskaya et al., 2017), cervix growth during pregnancy in rats and pigs (Burger & Sherwood, 1998; Huang, Li, & Anderson, 1997), growth of vagina and uterus in pregnant pigs (Min, Hartzog, Jennings, Winn, & Sherwood, 1997), new blood vessel formation and endometrial connective tissue maintenance in early pregnancy of rhesus monkeys (Goldsmith et al., 2004), and improvement of spermatozoan motility (Lessing et al., 1986).

The relaxin/RXFP1 system has been associated with cAMP, PI3K/Akt, NO/cGMP, MAPK and ERK1/2 signaling (reviewed in Valkovic, Bathgate, Samuel, & Kocan, 2019) (Valkovic et al., 2019). Binding of relaxins to their receptors recruits G‐proteins with subsequent activation of adenylyl cyclase and elevation of cAMP (Bathgate et al., 2013). Activation of NF‐κB by a cAMP/protein kinase A‐dependent mechanism may promote NOS2 (iNOS) expression and nitric oxide (NO) (Bani et al., 1998; Failli et al., 2002). NO has been shown to inhibit profibrotic TGFβ signaling by blocking phosphorylation of Smad2 (Heeg et al., 2005). PI3K/Akt‐associated signaling pathways can be activated by relaxin/RXFP1 to provide vasodilation in the cardiovascular system and regulate cell differentiation (Boccalini, Sassoli, Bani, & Nistri, 2018).

## 3 PROTEIN STRUCTURE AND FUNCTIONAL CHARACTERISTICS OF RXFP1

While much is known about the cell signaling pathways activated by relaxin, it is clear that ligand–receptor interactions are multidimensional and represent a potential site for cell signaling regulation. In experimental binding assays, relaxin dose, treatment length, and assay temperature contributed to the efficiency of relaxin binding to its receptor (Svendsen et al., 2009; Svendsen, Zalesko, et al., 2008). Cellular pH levels control ligand/receptor complex stability and play an important role in modulating the downstream signaling pathway (Svendsen, Zalesko, et al., 2008). Heterodimerization of WT RXFP1 with its splicing variants or other receptors exhibit negative cooperativity in relaxin/RXFP1 binding (Kern et al., 2008; Svendsen, Zalesko, et al., 2008). Furthermore, overexpression of RXFP1 in HEK‐293T cells resulted in its intracellular accumulation and inhibition of relaxin/RXFP1 signaling (Hoare et al., 2019). Crosstalk between RXFP1 and other receptors is also a current focus in RXFP1 research (Valkovic et al., 2019).

Like other GPCRs, RXFP1 protein consists of three major regions: the extracellular (EC), the transmembrane (TM), and the intracellular (IC) regions (Venkatakrishnan et al., 2013).

### 3.1 EC region

The EC region of RXFP1 consists of an N‐terminus and three extracellular loops (ECL1–ECL3) (Venkatakrishnan et al., 2013). ECLs link TM segments and contribute to ligand binding, TM positioning, and activation of GPCRs (Palczewski et al., 2000; Wheatley et al., 2012). Three protein domains are identified in the EC region: an LDLa module, a linker domain, and a LRR domain (Hoare et al., 2019).

The LDLa was first described and characterized in LDL receptor and was subsequently identified in other proteins with diverse biological functions (Brown & Goldstein, 1986; Hopkins, Bathgate, & Gooley, 2005). It contains three disulfide bonds and requires a bound calcium ion for its correct folding and stabilization (Hopkins et al., 2005). Although relaxin does not bind to LDLa directly, the binding of relaxin to RXFP1 stabilizes the LDLa/linker structure that leads to the direct contact between the EC and TM region (Diepenhorst et al., 2014; Hoare et al., 2019; Sethi et al., 2016). Removing LDLa module from RXFP1 abolished the ligand‐activated receptor signaling (Scott et al., 2006). Mutagenesis introduced in the LDLa module altered its native three‐dimensional structure (Hopkins et al., 2005; Koduri & Blacklow, 2001; Varret et al., 1997) and fully disrupted receptor activity (Hopkins, Layfield, Ferraro, Bathgate, & Gooley, 2007).

There is a 32‐residue linker hitching the N‐terminus LDLa module and the LRR domain together in RXFP1 (Sethi et al., 2016). This helically shaped linker provides a binding site essential for the steady binding of the relaxin A‐chain (Scott et al., 2006; Sethi et al., 2016). Although mutations in the linker residues have not been shown to affect receptor trafficking or G‐protein coupling, profound effects on reducing relaxin binding and decreasing cAMP response were observed (Sethi et al., 2016).

Different from most of the class A GPCR ligands that bind to the TM region directly, relaxin binds to the RXFP1 through the primary ligand binding site in the LRR domain of the EC region (Hoare et al., 2019). A shallow curvature structure formed by the 10 LRRs is predicted to serve as the primary high‐affinity relaxin binding site (Petrie, Lagaida, Sethi, Bathgate, & Gooley, 2015). The LRR domain potentially interacts with the linker after relaxin binding (Petrie et al., 2015; Scott et al., 2006). When relaxin binds to the LRR, it induces a conformational change of the receptor to position the LDLa module for interacting with the TM region (Hopkins et al., 2007).

### 3.2 TM region

In addition to the high‐affinity binding site in the EC region, there is a low‐affinity relaxin binding site in the TM region of RXFP1 (Halls et al., 2005). The TM regions of GPCRs form the main structural core of the receptor with seven α‐helices (TM1–TM7) folded together. Conformational changes of different TMs are important for transducing the ligand/receptor interaction to the IC region (Venkatakrishnan et al., 2013). Mutations in the TM region affected receptor conformational selectivity and ligand‐binding affinity in vitro (Dore et al., 2011; Heitz et al., 1999). Two single amino acid changes in the TM6 resulted in dose‐dependent increases of cAMP production (Hsu et al., 2000).

### 3.3 IC region

The IC region of GPCR interfaces with cytosolic signaling proteins. It includes three intracellular loops (ICL1–ICL3), an intracellular amphipathic helix, and a unique C‐terminal tail containing a phosphorylation site (Hsu et al., 2000; Scheerer et al., 2008). The C‐terminal half of ICL3 plays an important role in linking the relaxin‐activated RXFP1 receptor with G protein (Shpakov et al., 2007).

In summary, activation of RXFP1 by relaxin is a complex multistep process. Relaxin binding initiates RXFP1 signaling. However, the completion of RXFP1‐dependent signal transduction requires the interactions between different receptor regions and correct conformation of the ligand/receptor complex to initiate downstream IC signaling (Sethi et al., 2016).

## 4 ALTERNATIVE SPLICING VARIANTS AND RXFP1 FUNCTION

**Figure 1:**
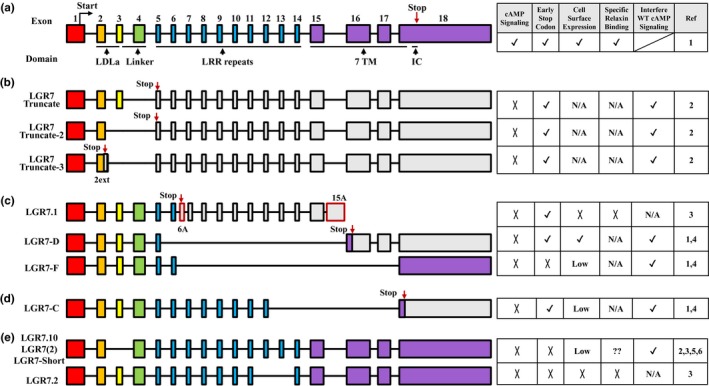
Alternative splicing variants of RXFP1. The genomic structures are shown on the left. The functions of each splicing variant in relaxin binding, signaling, and interfering of wild‐type RXFP1 function are shown on the right. The designations from original reports for each alternative splicing variant are shown. Coding exons are shown in colors and noncoding exons are shown as gray boxes. The locations of novel premature stop codons are shown. (a) Wild‐type RXFP1 gene. Only exons were drawn based on their relative size. The coding exons for each protein domains are shown. (b) Genomic structures of three truncated N‐terminus RXFP1 splicing variants that retain the LDLa module. (c) Genomic structures of truncated N‐terminus RXFP1 that retain both LDLa module and linker domain. For the novel exons 6A and 15A in LGR7 are shown in red‐framed boxes. (d) Genomic structure of a truncated N‐terminus RXFP1 splicing variant, LGR7‐C, retains LDLa module, linker domain and majority of LRRs. (e) Genomic structures of two splicing variants resulted from in‐frame deletion. For the summary table, positive function is labeled as (✓), lack of function is labeled as (X), inconclusive findings in the literature is labeled as (??), and not analyzed is labeled as (N/A). Ref, cited references are: 1. Kern et al. (2008); 2. Scott et al. (2006); 3. Muda et al. (2005); 4. Kern and Bryant‐Greenwood (2009); 5. Hsu et al. (2000); 6. Scott et al. (2006). Abbreviations: LDLa, low‐density lipoprotein class A; LRR, leucine‐rich repeat

The *RXFP1* gene is localized on chromosome 4 with 18 exons and encodes a protein with 757 amino acids (Figure 1a) (Hsu et al., 2000). *RXFP1* mRNA is detectable in testis, ovary, adrenal gland, uterus, small intestine, colon, kidney, brain, endometrium, lung, heart, and placenta (https://www.ncbi.nlm.nih.gov/gene/59350) (Fagerberg et al., 2014; Hsu et al., 2000). Interestingly, multiple smaller *RXFP1* transcripts were detected in different tissues suggesting the presence of alternative splicing (Hsu et al., 2000). As many as 29 alternative splicing variants have been identified, and 9 of them have been characterized in detail (Hsu et al., 2000; Kern & Bryant‐Greenwood, 2009; Kern et al., 2008; Muda et al., 2005; Scott, Fu, et al., 2005; Scott et al., 2006). Figure 1 summarizes the skipped exons, corresponding protein regions, and known functional consequences for these characterized RXFP1 variants.

### 4.1 Truncated N‐terminus RXFP1 retaining the LDLa module

Three splicing variants that encode truncated RXFP1 proteins retaining the N‐terminus LDLa module are identified in human uterus tissue (Figure 1b) (Scott et al., 2006). Since RXFP1 was initially named as LGR7, alternative splicing variants for RXFP1 were all designated based on the old nomenclature. These include one exon 4 skipping (designated as *LGR7‐Truncate*), one exon 3 and exon 4 skipping (*LGR7‐Truncate 2*) and one exon 3 and exon 4 skipping with extended intronic sequences attached to the end of exon 2 (*LGR7‐Truncate 3*) (Scott et al., 2006; Scott, Tregear, et al., 2005). All three splicing variants result in open reading frameshifts and a premature stop codon in exon 5 (*LGR7‐Truncate* and *LGR7‐Truncate 2*) and a new extended exon 2 (*LGR7‐Truncate 3*). Additional amino acids, ranging from 1 to 7, are attached to the C‐terminus of these truncated RXFP1 proteins (Scott et al., 2006). Functional analysis of the LGR7‐Truncate showed no interference with surface expression of the wild‐type (WT) RXFP1 co‐expressed in HEK‐293T cells (Scott et al., 2006). In contrast, LGR7‐Truncate inhibited cAMP accumulation induced by WT RXFP1 dose dependently (Scott et al., 2006; Scott, Tregear, et al., 2005). Interestingly, the naturally occurring splicing variant partially lacking the LDLa module (*LGR7.10* in Figure 1e) results in normal relaxin binding but abolished its ability of inducing cAMP accumulation (Scott et al., 2006). In mouse, the *LGR7‐Truncate* is expressed in pregnant uterus and not in brain (Scott et al., 2006). These studies suggest that the LDLa module may act as an antagonist affecting cAMP accumulation and may differentially regulate relaxin/RXFP1 signaling during pregnancy and delivery.

### 4.2 Truncated N‐terminus RXFP1 retaining both LDLa module and linker domain

In contrast to the splicing variants that retain the LDLa module but lack the linker domain, three of the known splicing variants encoded truncated RXFP1 proteins retain both the LDLa module and the linker domains (Figure 1c) (Kern et al., 2008; Muda et al., 2005). These variants were identified initially in the human fetal membrane and placental tissues and encode RXFP1 proteins lacking the majority of the LRR domain, the TM, and the IC regions (Kern et al., 2008; Muda et al., 2005). One of them (*LGR7.1*) has two novel exons after exon 6 (exon 6A) and exon 15 (15A) (Muda et al., 2005). Exon 15A contains an alternative poly‐A signal while 6A has a premature stop codon (Muda et al., 2005). *LGR7.1* is translated into a RXFP1 protein containing the N‐terminus region with only two LRRs and 10 nonhomologous amino acids at the C‐terminus of the truncated protein (Muda et al., 2005). Kern and colleagues have also identified and characterized two splicing variants (*LGR7‐D* and *LGR7‐F*) that encode similarly truncated RXFP1 proteins as *LGR7.1* (Kern & Bryant‐Greenwood, 2009; Kern et al., 2008). The LGR7‐D protein is encoded by a splicing variant lacking exon 6 through exon 15 generated by cryptic splice sites and contains the LDLa module, one LRR, and 25 nonhomologous amino acids (Kern et al., 2008). The *LGR7‐F* is a result of an alternative splicing of exon 6 to exon 18 with cryptic splicing sequences (Kern et al., 2008). It encodes a RXFP1 protein containing the N‐terminus part up to and including the first two LRRs and 10 nonhomologous amino acids at the end (Kern et al., 2008). The LGR7.1 is expressed in different tissues (Muda et al., 2005). Direct comparison of expression levels in the placenta demonstrated much lower levels of the *LGR7‐D* and *LGR7‐F* compared to the *WT RXFP1* (Kern et al., 2008). Functional analysis revealed that the LGR7.1 and LGR7‐F are predominantly retained within cells (Kern et al., 2008; Muda et al., 2005), while the LGR7‐D is expressed intracellularly and on the cell surface (Kern et al., 2008). When co‐expressed with WT in HEK‐293 cells, both LGR7‐D and LGR7‐F colocalized with WT RXFP1 within the cells and reduce RXFP1‐mediated cAMP accumulation (Kern et al., 2008). In addition, these two truncated variants have dominantly negative effects in WT RXFP1 maturation, homodimerization in the endoplasmic reticulum, and cell surface expression (Kern et al., 2008).

### 4.3 Truncated N‐terminus RXFP1 retaining LDLa module, linker domain, and majority of LRRs

Another splicing variant (*LRP7‐C*) misses the TM and IC regions but retains 8 of the 10 LRRs (Figure 1d) (Kern et al., 2008). The *LGR7‐C* is a result of alternative splicing between exon 12 and exon 18 that creates a novel stop codon at the beginning of exon 18 (Kern et al., 2008). Although it contains 8 LRRs, the LGR7‐C is mainly retained inside the cells and has a similar function as the three truncated N‐terminus RXFP1 retaining only 1 or 2 LRRs (Figure 1c).

### 4.4 RXFP1 variants with in‐frame deletions

Two splicing variants of RXFP1 result from in‐frame deletions and have been characterized (Figure 1e) (Hsu et al., 2000; Muda et al., 2005). One variant skips exon 3 [*LGR7.10* based on Muda et al. (2005), *LGR7(2)* based on Hsu et al. (2000) or *LGR7‐Short* based on Scott et al. (2006), Scott, Tregear, et al. (2005) and will be referred as *LGR7.10* in this review] and the other skips both exon 12 and 13 (*LGR7.2*) which is different from the *LGR7(2)* mentioned above (Hsu et al., 2000) (Muda et al., 2005). The *LGR7.10* is detected in the ovary, pituitary, placental, prostate, and uterus tissues and encodes a RXFP1 with an in‐frame deletion of the linker region (Hsu et al., 2000; Muda et al., 2005). When *LGR7.10* or *LGR7.2* are overexpressed in HEK‐293T cells, only the LGR7.10 was detected on the cell surface but at a very lower level compared to the WT (Muda et al., 2005). The LGR7.2 lost its responsiveness to relaxin and relaxin binding (Muda et al., 2005). Specific binding of relaxin to the LGR7.10 was not detected in a study reported by Muda et al., however, a later study demonstrated specific relaxin binding to this RXFP1 variant in HEK‐293T cells (Muda et al., 2005; Scott et al., 2006).

In summary, all characterized RXFP1 splicing variants have been shown to lose their ability to activate relaxin‐dependent cAMP accumulation. Seven of the nine splicing variants have been shown to interfere with cAMP accumulation mediated by WT RXFP1 signaling. Given the large size of the *RXFP1* gene and the numbers of coding exons, we speculate that tissue‐specific splicing variants will be discovered in the future. The differential tissue expression and antagonistic (dominant‐negative) function of these splicing variant receptors suggest that complex posttranscriptional regulation of RXFP1 gene may play important roles in spatial and temporal expression and signaling of relaxin/RXFP1 (Halls, van der Westhuizen, Bathgate, & Summers, 2007).

## 5 RXFP1 AND CANCER

Studies related to relaxin/RXFP1 and human diseases have been centered on cancer and fibrotic diseases. Relaxin/RXFP1‐mediated cancer growth and invasion have been reported in breast, thyroid, prostate, and other cancer models (Bigazzi, Brandi, Bani, & Sacchi, 1992; Binder, Hagemann, Husen, Schulz, & Einspanier, 2002; Feng et al., 2007; Hombach‐Klonisch et al., 2006; Hombach‐Klonisch, Buchmann, Sarun, Fischer, & Klonisch, 2000; Tashima, Mazoujian, & Bryant‐Greenwood, 1994; Vinall et al., 2011). In most of these cancers, relaxin and relaxin‐like peptides are overexpressed and exert their effects by activating different signaling cascades (Bigazzi et al., 1992) (Hombach‐Klonisch et al., 2000; Tashima et al., 1994) (Vinall et al., 2011). Although the role of RXFP1 in cancer has not been fully understood, it has emerged as a therapeutic target to reverse the procancer effects of increased relaxin (recently reviewed by Thanasupawat et al., 2019) (Thanasupawat et al., 2019). Downregulation of RXFP1 in prostate cancer cells decreased tumor formation induced by these cells in nude mice (Feng et al., 2010).

Are splice variants associated with disease states? Overexpression of LDLa module of RXFP1 in prostate cancer cells resulted in a decrease in proliferation, soft agar colony formation, adhesion and invasion in vitro, and tumor growth in mouse model (Feng & Agoulnik, 2011). These findings suggest that alternative splicing variants retaining different domains of the RXFP1 protein may modulate relaxin function in cancer. In addition, the formation of both GPCR homodimer and heterodimer contributes to the complexity of GPCR signaling (Angers, Salahpour, & Bouvier, 2002). RXFP1 forms a homodimer when it is transported from the ER to the cell membrane and negative cooperativity occurs when it forms a heterodimer with RXFP2 (Svendsen, Vrecl, et al., 2008; Svendsen et al., 2009; Svendsen, Zalesko, et al., 2008). Therefore, dimerization with other receptors or RXFP1 splicing variants may play important roles in normal RXFP1 function and in diseases.

## 6 RXFP1 AND FIBROTIC DISEASE

Strong preclinical studies support relaxin as a potent antifibrotic molecule (Lam, Royce, Samuel, & Bourke, 2018; McVicker & Bennett, 2017; Ng, Leo, Parry, & Ritchie, 2018; Pini et al., 2010; Samuel, 2005; Samuel et al., 2017; Sasser, 2013). However, relaxin‐based clinical trials failed to show any therapeutic effects in patients with systemic sclerosis (SSc or scleroderma) (Casten & Boucek, 1958; Jefferis & Dixon, 1962; Khanna et al., 2009). Emerging studies demonstrate that the unresponsiveness to relaxin‐based therapy is due to the downregulation of RXFP1 expression in fibrotic tissues, such as lung and skin (Bahudhanapati et al., 2019; Corallo et al., 2019; Giordano et al., 2012; Tan et al., 2016). Therefore, RXFP1 becomes a therapeutic target to restore the responsiveness of fibrotic tissues (Bathgate et al., 2018). Table 1 summarized studies on relaxin and RXFP1 expressions in different fibrotic diseases.

**Table 1:** Tissue‐specific expression of relaxin and RXFP1 in fibrotic diseases

Disease	Tissue/cell	Relaxin		RXFP1		Reference
		mRNA	Protein	mRNA	Protein	
SSc	Skin				↓	Giordano et al. (2012)
	Skin fibroblast				↓	Giordano et al. (2012)
	Skin fibroblast			↑		Corallo et al. (2019)
	Lung				↓	Tan et al. (2016)
	Lung fibroblast				↓	Tan et al. (2016)
	Blood		↑			Giordano et al. (2005)
IPF	Lung tissue	↑		↓	↓	Tan et al. (2016)
	Lung fibroblast			↓	↓	Tan et al. (2016)
	Lung fibroblast			↓	↓	Bahudhanapati et al. (2019)
ESRD	Blood		↑ with death			Hocher et al. (2004)
CHF	Blood		↑			Han et al. (2017)
AMI	Blood		↑			Zhang et al. (2015)
AHF	Blood		↑ with severity			Pintalhao et al. (2017)
HF	Blood		↓			Kupari et al. (2005)
AF	Blood		↑ in patients with recurrence			Qu et al. (2019)
	Blood		↑ in HF			Zhou et al. (2016)
HT	Blood		↓			Gedikli et al. (2009)
Liver Cirrhosis	Liver			↑		Nagorniewicz et al. (2019)
	Liver				↑	Fallowfield et al. (2014)

Note

The changes in RXFP1 expression are indicated by up or down arrows.

Abbreviations: AF, atrial fibrillation; AHF, acute heart failure; AMI, acute myocardial infarction; CHF, congestive heart failure; ESRD, end‐stage renal disease; HF, heart failure; HT, hypertension; IPF, idiopathic pulmonary fibrosis; RXFP1, relaxin family peptide receptor 1; SSc, systemic sclerosis.

### 6.1 Lung and skin fibrosis

SSc is a group of heterogeneous disorders characterized by varying degrees of fibrosis of the skin and internal organs (Haustein, 2002; Silman, 1997). Lung fibrosis is one of the most common manifestations and is a major cause of SSc‐related mortality (Denton, Wells, & Coghlan, 2018). The protective role of relaxin signaling in lung fibrosis has been demonstrated in relaxin knockout mice (Samuel et al., 2005; Unemori et al., 1996). Similarly, the RXFP1‐null mice develop early onset peribronchiolar and perivascular fibrosis compared to the relaxin‐null mice (Kamat et al., 2004). Relaxin has been tested in SSc patients as early as 1958 with no beneficial effects (Casten & Boucek, 1958; Jefferis & Dixon, 1962). A smaller study with relaxin showed some efficacy in reducing skin fibrosis (Seibold et al., 2000) which was not validated in a large clinical trial with SSc patients (Khanna et al., 2009).

RXFP1 protein expression in fibrotic lung and skin of SSc patients is dramatically reduced. RXFP1 is similarly downregulated in SSc lung and skin fibroblasts (Corallo et al., 2019; Giordano et al., 2012; Tan et al., 2016). Increased relaxin in peripheral blood was also reported in SSc patients (Giordano et al., 2005). However, the relative reduction of RXFP1 expression in fibrotic tissues may potentially render these tissues insensitive to relaxin. Interestingly, bulk RNA sequencing of SSc skin fibroblasts detected upregulation of 13 different mRNA isoforms without detectable expression of RXFP1 protein in these cells (Corallo et al., 2019). This study supports that the splicing variants of RXFP1 may be important regulators of RXFP1 expression in different fibrotic diseases.

Idiopathic pulmonary fibrosis (IPF) is a progressive disease with an average survival of 2.5 years (King, Pardo, & Selman, 2011). Patients with IPF or other forms of interstitial lung disease may have better pulmonary function if their lung‐specific *RXFP1* expression is higher (Tan et al., 2016). In the bleomycin lung fibrosis mouse model, treating with a relaxin‐like agonist reduced bleomycin‐induced collagen deposition in vivo (Pini et al., 2010; Tan et al., 2016). Most notable, RXFP1 expression is dramatically decreased in both lung tissues and lung fibroblasts of IPF patients (Tan et al., 2016). In vitro, silencing of RXFP1 expression was associated with insensitivity to exogenous relaxin, which could be reversed by enhancement of RXFP1 expression in IPF lung fibroblasts (Tan et al., 2016). The findings in both SSc and IPF support that the lack of or reduced expression of RXFP1 in fibrotic tissues of IPF and SSc contributes to the failed responses to relaxin for IPF lung fibroblasts in vitro and relaxin‐based therapies in SSc clinical trials (Casten & Boucek, 1958; Jefferis & Dixon, 1962; Khanna et al., 2009; Tan et al., 2016).

What drives downregulation of RXFP1? Reduction of *RXFP1* mRNA suggests that transcriptional mechanisms may account for this. TGFβ decreases expression of *RXFP1* at the level of mRNA (Bahudhanapati et al., 2019; Corallo et al., 2019; Tan et al., 2016). Our group recently reported that microRNA‐144‐3p (miR‐144‐3p) regulates RXFP1 in fibrotic lung fibroblasts (Bahudhanapati et al., 2019). MiR‐144‐3p is upregulated in IPF fibroblasts compared with control donor lung fibroblasts. Overexpression of a miR144‐3p mimic and anti‐miR144‐3p in the donor lung fibroblasts resulted in the down‐ and upregulation RXFP1, respectively. Interestingly, Yong and colleagues have also demonstrated that knocking down RXFP1 gene by a synthetic microRNA resulted in a loss of relaxin responsiveness of human dermal fibroblasts (Yong, Callander, Bergin, Samuel, & Bathgate, 2013). In addition to the micro‐RNA regulation, *RXFP1* may be regulated by transcription factors important in fibrotic diseases. Therefore, abnormal regulation of RXFP1 expression in fibrotic lung and skin tissues is a therapeutic target for reversing tissue fibrosis.

### 6.2 Kidney fibrosis

Relaxin has been reported as a natural protective agent against induced or age‐related renal fibrosis (Samuel et al., 2004). Relaxin treatment in an animal model of kidney disease decreases serum creatinine, proteinuria, and interstitial fibrosis (McDonald et al., 2003). Mice lacking relaxin experienced more kidney interstitial fibrosis (Hewitson et al., 2007). Interestingly, in patients with end‐stage renal disease, higher levels of circulating relaxin are associated with mortality (Hocher et al., 2004) although the status of renal‐specific RXFP1 expression in these patients is not known. Short time infusion of serelaxin in patients with alcohol‐related liver cirrhosis increases renal blood flow and decreases renal vascular resistance (Snowdon et al., 2017). Relaxin/RXFP1 signals through pERK1/2 and a neuronal nitric oxide (nNOS)‐NO‐sGC‐cGMP‐dependent pathway to inhibit TGF‐β1/Smad2 pathway and to reduce renal myofibroblast differentiation (Chow et al., 2012; Heeg et al., 2005; Mookerjee et al., 2009). Both angiotensin II type 1 receptor (AT_1_R) and type 2 receptor (AT_2_R) can form a heterodimer with RXFP1 (Chow et al., 2014; Chow et al., 2019; Sasser, 2013, 2014). Antagonists for either AT_1_R or AT_2_R block relaxin/RXFP1 signaling, supporting comprehensive crosstalk between RXFP1 homodimer and RXFP1‐AT_1_R and RXFP1‐AT_2_R heterodimers (Chow et al., 2019). In addition, AT_2_R activation reduces TGF‐β1 stimulation of profibrotic pathways (Jones, Vinh, McCarthy, Gaspari, & Widdop, 2008; Peluso, Santos, Unger, & Steckelings, 2017; Wang et al., 2017). The complexity of RXFP1 homo and heterodimerization suggests that RXFP1 is at the center of renal‐specific relaxin/RXFP1 antifibrotic signaling.

### 6.3 Cardiac fibrosis

Relaxin was also introduced as a cardioprotective factor in ischemic heart diseases, atrial fibrillation, and cardiac remodeling in aged heart (Henry et al., 2016; Martin, Romero, & Salama, 2019; Parikh et al., 2013; Zhang et al., 2005). Treatment with relaxin specifically reverses cardiac fibrosis in the cardiomyopathy or hypertrophy fibrosis animal models (Bathgate et al., 2008; Parikh et al., 2013; Sun et al., 2019). Several reviews focusing on the relaxin and their protective roles in cardiac fibrosis have published in recent years (Barker, Tan, & Clevers, 2013; Devarakonda & Salloum, 2018; Du, Bathgate, Samuel, Dart, & Summers, 2010; MacLean & Pasumarthi, 2014; Martin et al., 2019; Ng et al., 2018; Sarwar, Du, Dschietzig, & Summers, 2017; van der Westhuizen et al., 2008). Lower levels of circulating relaxin were reported in patients with heart failure and hypertension (Gedikli et al., 2009; Kupari, Mikkola, Turto, & Lommi, 2005). However, there are multiple studies that reported increases of peripheral blood relaxin and positive correlation of relaxin levels with disease severity in cardiovascular diseases (Han et al., 2017; Pintalhao et al., 2017; Qu et al., 2019; Zhang et al., 2015; Zhou et al., 2016). It will be important to determine the tissue‐specific relaxin and RXFP1 expression in the affected cardiac tissues for understanding the complexity of cardiac‐specific relaxin/RXFP1 signaling. The RXFP1‐AT_1_R and RXFP1‐AT_2_R heterodimers described in kidney fibrosis also play important roles in the cardiac system (Chow et al., 2014; Chow et al., 2019; Sasser, 2014). A nonpeptide‐based small molecule relaxin mimetic, ML290, exerts its antifibrotic effects by inhibiting TGF‐β1‐induced Smad2 and Smad3 phosphorylation and increasing matrix metalloprotease 2 expression (Kocan et al., 2017). In addition, a relaxin B‐chain‐only analog, B7‐33, has strong antifibrotic effects in multiple rodent cardiac fibrosis models by activating RXFP1‐AT_1_R and RXFP1‐AT_2_R heterodimers and downstream signaling (Barker et al., 2013; Chow et al., 2019; Hossain et al., 2016).

### 6.4 Liver fibrosis

The major cause of liver fibrosis is the over activation of hepatic stellate cells and their transformation into myofibroblast‐like cells after liver damage (Williams et al., 2001). In the rat carbon tetrachloride model, relaxin increases intrahepatic NO level and reduces hepatic expression of profibrotic markers and portal pressure (Fallowfield et al., 2014). A phase II randomized open‐label clinical study of serelaxin in patients with alcohol‐related liver cirrhosis and portal hypertension was reported (Snowdon et al., 2017). The small molecule relaxin agonist, ML290, also shows antifibrotic effects in an in vitro liver organoid model and an in vivo liver fibrosis mouse model (Kaftanovskaya et al., 2019). Interestingly, unlike skin and lung fibrosis, dramatically increased hepatic expression of *RXFP1* has been observed in a rat model of liver cirrhosis and—in contrast to lung and skin‐‐*higher* expression of RXFP1 is correlated with increased liver fibrosis in human (Fallowfield et al., 2014; McBride et al., 2017; Nagorniewicz et al., 2019). However, whether the upregulation RXFP1 is related to the alternative splicing transcripts or protein variants is not known. The regulation of RXFP1 in liver fibrosis may be fundamentally different from that in lung, skin, and other fibrotic organs.

## 7 CONCLUSIONS

Relaxin/RXFP1 signaling is important in both normal physiology and in human diseases. Reduced expression of RXFP1 in fibrotic lung and skin tissues surrenders both relaxin/RXFP1 signaling and their responsiveness to exogenous relaxin treatments. Several questions remain. These include how splice variants of RXFP1 regulate expression and relaxin sensitivity. Understanding the molecular mechanisms that drive aberrant expression of RXFP1 in disease will provide insight into therapeutic targets that may restore the relaxin responsiveness of fibrotic tissues.
